# Depression in Patients with Alcohol Dependence Syndrome in a Tertiary Care Center: A Descriptive Cross-sectional Study

**DOI:** 10.31729/jnma.6967

**Published:** 2021-08-31

**Authors:** Mukti Acharya

**Affiliations:** 1 Department of Psychiatry, Nobel Medical College and Teaching Hospital, Biratnagar, Nepal

**Keywords:** *alcohol dependence*, *comorbidity*, *depression*

## Abstract

**Introduction:**

Alcohol dependence syndrome is an important and major public health issue seen in our community and health center. It is mostly associated with different mental health problems and psychiatric co-morbidities. This study aims to find out the prevalence of depression among alcohol dependence syndrome in a tertiary care hospital.

**Methods:**

A descriptive cross-sectional study conducted over one year from April 15, 2020, to February 15, 2021 at a tertiary care hospital. The ethical approval was obtained from the Institutional Review Committee of Nobel Medical College (reference number: 409/2020). Convenience sampling method was used. The patients admitted for alcohol use and related problems in the Psychiatry Department, Nobel Medical College Teaching Hospital, Biratnagar were included. Data entry was done using Microsoft Excel and statistical analysis was done by using Statistical Packages of Social Sciences Version 16.0. Point estimate at 95% Confidence Interval was calculated along with frequency and percentage.

**Results:**

The prevalence of depression in patients with Alcohol Dependence Syndrome was 17 (16.3%) (95% Confidence Interval= 9.20-23.39) . The patients suffering from depressive disorder male and female were 16 (94.1%) and 1 (5.8%) respectively. The participants with Alcohol Dependence Syndrome with no other comorbid psychiatric disorders were found to be 60 (57.7%).

**Conclusions:**

Psychiatric comorbidity found to be common in alcohol dependent people among which depression was found to be most common.

## INTRODUCTION

Depression is the condition when the individual usually suffers from depressed mood, loss of interest and enjoyment, and reduced energy leading to increased fatigability and diminished activity.^1^ Similarly, Alcohol dependence syndrome (ADS) is defined by the International Classification of Diseases as the presence of three or more indicators of dependence for at least a month within the previous year.^1^

Several studies have shown that the high correlation has been tightly coupled with psychiatric comorbidity and alcohol.^2-6^ Given the fact that Psychiatry and people's acceptance to visit Psychiatry related treatment or refer to treat patients still lies to reluctant compared to physical medical treatment. While the developed countries have been heavily focused in academics, research and resources for such study-Nepal remains one of the underdeveloped countries to be lacking adequate study in Alcohol Dependence Syndrome and comorbidities.

The aim of the study is to find the prevalence of depression in the Eastern Terai region of Nepal with the provided tertiary care setup.

## METHODS

A descriptive cross-sectional study was conducted over the period of one year from April 15, 2020, to Feb 15, 2021. The ethical approval was obtained from Nobel Medical College IRC (Dated April 15, 2020, IRC-NMCTH-409/2020). Sample size as calculated using the formula-

n = Z^2^ × p × q / e^2^

  = (1.646)^2^ × 0.5 × (1-0.5) / (0.1)^2^

  = 68

Where,

n = sample size,
Z = 1.96 at 95% Confidence Interval (CI),
p = prevalence taken as 50% for maximum sample size
q = i-p
e = margin of error, 10%

The sample size calculated was sixty eight. The sampling was done by convenience sampling method. The study set up was based on Nobel Medical College Psychiatry in-patient department who were admitted for alcohol use disorder and related problems. The participants who were included were the patients who met the diagnosis for alcohol dependence. Those who were uncooperative and did not give consent were excluded from the study group. Written consent was obtained from participants. The details of socio-demographic profiles and clinical variables were noted. MINI 6.0 scale was applied to all the patients with alcohol use disorder to see the depressive disorder.^7,8^ Those who had positive depressive disorders in MINI 6.0 were then applied to the ICD-10 (International classification of diseases, clinical description and diagnostic guidelines) and final diagnosis was considered.^1^ The data entry was maintained in Excel. The detailed analysis of data is provided in the 'results' section which includes the frequency, relationship map and breakdown by several variables. IBM Statistical Package for the Social Sciences Version 16.0 is used for data analysis. Point estimate at 95% Confidence Interval was calculated along with frequency and percentage for binary data.

## RESULTS

The conducted study showed the prevalence of depression 17 (16.3%) (95% Confidence Interval= 9.20-23.39) in alcohol dependence patients. ADS with no other comorbid psychiatric disorders were found to be 60 (57.7%), other comorbid psychiatric Diagnosis by ICD-10, Psychosis 13 (12.5%), anxiety 5 (4.8%), Seizure disorder 5 (4.8%), suicidal attempts 3 (2.9%) and OCD 1 (0.9%) (Table 1,2). In the study, out of total patients, comorbid physical illnesses were not found to be present in 78 (75%) patients. The comorbid physical illness found were CLD in 10 (9.6%) patients, Acute Pancreatitis, Alcoholic Cardiomyopathy, Essential Tremor, Fatty Liver, Hypertension, Hypertension with Diabetes Mellitus, LRTI, Rheumatoid Arthritis, UTI and Esophageal Candidiasis resulted to single number of patients.

**Table 1:** Patients by comorbid psychiatric diagnosis (ICD-10).

Types	n (%)
ADS Only (No comorbid psychiatric disease)	60 (57.7)
Depression	17 (16.3)
Other psychiatric diseases	27 (25.9)
Total	104 (100)

**Table 2:** Other co-morbid psychiatric diagnoses (ICD-10).

Types	n (%)
Psychosis	13 (12.5)
Anxiety	5 (4.8)
Seizure Disorder	5 (4.8)
Suicidal Attempt	3 (2.8)
OCD (Obsessive compulsive disorder)	1 (0.9)

In the conducted study participants suffering from depressive disorders male and female were 16 (94.1%) and 1 (5.8%) respectively. The total participant in the study group male comprised 92 (88.5%), and female of 12 (11.5%). Sixteen (94.1%) were married and 1 (5.8%) was an unmarried patient with depressive disorder out of the total participant 94 (90.4%) and unmarried 10 (9.6%). There was patients with depressive illness 14 (82.3%) were from urban and 3 (17.6%) from rural area out of the total participants 58 (55.8%) from urban and 46 (44.2%) from rural area. Patients suffering from depression all 17 (16.3%) were Hindus. Breaking down by religion among the total participants, 94 patients (90.3%) were found from Hindu religion and rest 10 (9.6%) from mix of Muslim, Christianity, Buddhism and undecided. The division by Cast showed that depressive patients Janajati 5 (29.41%), Chhetri 4 (23.52%), Madhesi 4 (23.52%), Brahaman 3 (17.64%) and Dalit 1 (5.88%). Similarly, data breakdown by occupation of the patient with depressive disorder highest number were businessman 7 (41.17%) followed by farmer 3 (17.64%), unemployed 3 (17.64%), house wife 1(5.88%) and service holder 1 (5.88%). Among the total participants Farmer was found to be 39 (37.5%), second to be in the unemployed category of 23 (22.1%) followed by businessman being the third highest of 15 (15.4%) (Table 3).

**Table 3:** Socio demographic characteristics of patients with Depression (n=17).

Variables		n (%)
Gender	Male	16 (94.11)
	Female	1 (5.88)
Marital status	Married	16 (94.11)
	Unmarried	1 (5.88)
Residence	Urban	14 (82.35)
	Rural	3 (17.64)
Cast	Brahman	3 (17.64)
	Chettri	4 (23.52)
	Dalit	1(5.88)
	Janajati	5 (29.41)
	Madhesi	4 (23.52)
Education	Illiterate	3 (17.64)
	Literate	2 (11.76)
Primary		1 (5.88)
	Secondary	1 (5.88)
Higher secondary		6 (35.29)
	Graduate and above	4 (23.52)
Occupation	Businessman	7 (41.17)
	Farmer	3 (17.64)
	Housewife	1 (5.88)
	Service holder	1 (5.88)
	Student	2 (11.76)
	Unemployed	3 (17.64)

## DISCUSSION

Nobel medical college and teaching hospital is a tertiary care center to the mental health covering the eastern part of Nepal. This study aimed to observe the prevalence of depression in alcohol dependent patients attending the psychiatric inpatient department. In the current study, depression was found to be the most prevalent among 104 patients. Psychiatry comorbidity was found to be highest in 44 (42.3%) patients. This prevalence is similar to that of studies by Sharma B, et al.^6^ (45.16%) and Vohra AK, et al.^9^ (76.6%). Shakya DR, et al. found 18.7% mood disorders among alcohol use disorders.^10^ In accordance with our study, Sedain, et al.^11^ had 28.5% patients with comorbidity out of a total of 263 patients. A hospital based study by Ravikanth T, et al. also reported a similar prevalence of 33% had comorbid psychiatric disorders.^12^

In the current study, depression was found to be 16.3% as the highest comorbid psychiatric diagnosis whereas the studies done by Sharma B, et al.^6^ found highest comorbid Axis-I psychiatric diagnosis was anxiety disorders (35.71%) followed by depression (28.57%) whereas study done by Vohra AK, et al.^9^ showed that highest was Axis-I disorders and Shakya DR.^10^ et al. showed 18.7% was mood disorders. Sedain et al.^11^ showed that Psychosis is 12.36% followed by anxiety disorder 7.33% and depressive disorder 6.56% showing significant differences from our study. The prevalence of depression in the current study had similar findings with the study done by Ravikanth T, et al.^12^ The study done by Ravikanth T, et al.^12^ showed that the most common psychiatric comorbidity was mood disorder (18%) followed by anxiety disorder (11%) and psychotic disorder (4%) and among the mood disorders, the most common was MDD (8%).

In the conducted study there were 16 (94.11%) male and 1 (5.88%) female suffering from depressive illness, out of total study group population male comprise of 92 (88.5%), and female of 12 (11.5%) which showed there were relatively fewer number of female patients seeking medical help in eastern region of Nepal. Similar to that of the study done by Sharma B, et al.^6^ showed the highest participant were male (80.63%) and Sedain, et al.^11^ also showed that the predominant number were male (93.15%) participants which had similar findings to the current study. Study done by Sharma B, et al.^6^ showed married with comorbidity was 35.48% and without comorbidity was 51.61% whereas unmarried were few in number with comorbid diagnosis was 9.7% and without comorbid diagnosis was 3.22%. We had similar findings having the large married population. In the current study, total married and unmarried patients were found to be 94 (90.4%) and 10 (9.6%) respectively and depressive married and unmarried patients were 16 (94.11%) and 1 (5.88%) respectively. In our study, there were 46 (44.2%) of patients from rural areas and 58 (55.8%) from urban areas. Out of these depressive patients 14 (82.35%) were from urban areas and 3 (17.64%) were from rural areas. In the current study more participants were from urban areas. Whereas Vohra AK, et al.^9^ showed that patients with comorbid psychiatric diagnosis was 52.2% from rural area and 47.8% from urban area and patients with comorbid psychiatric diagnosis was 71.4% from rural and 28.6% from urban area. Breaking down by religion, all the depressive patients were Hindu 17 (16.3%) and among the total participants in the study group 94 (90.4%) patients were from Hindu religion and rest other 10 (9.6%) from mix of Muslim, Christianity, Buddhism and undecided. The maximum numbers of the participants were Hindu as Nepal's predominant population was Hindus so there was not significant finding in correlation with religion. In our study among the depressive patients Janajati 5 (29.41%) was highest prevalence followed by Chhetri 4 (23.52%), Madhesi 4 (23.52%), Brahamin 3 (17.64%) and Dalit 1(5.88%). The total participants data result by Cast patients from Janajati being the highest percentage of 47 (45.19%) and second highest from Brahman 20 (19.23%) which is different from the study done by Sedain et al.^11^ highest number of cases were of lower caste, Baisya 52.85% followed by Sudra 21.67%. Similarly data breakdown by occupation the most common depressive patients were Businessman 7 (41.17%) by occupation. Among the total participants data breakdown by occupation Farmer were found to be 39 (37.5%), second to be in unemployed category were 23 (22.1%) followed by businessman being the third highest 15 (15.4%) which is approximate in comparison to study done by Sharma B, et al.^6^ Sharma B, et al.^6^ showed more participant with comorbid diagnosis were employed (41.93%) and few were unemployed (3.22%) whereas without comorbid psychiatric diagnosis showed that employed were 38.71% and unemployed was 16.2%. Hence occupation or employment status of the patient showed significant importance in understanding the comorbid alcohol dependence syndrome.

This study suggested a significant prevalence of depression and other comorbid psychiatric diagnosis in alcohol dependent patients.

The limitation of the study was that the sample size of females was small compared to male (92). This also included the patients who had a consent form signed by the patient and immediate relatives to be released. The generalization of the study cannot be applied to another demographic region as it is a small-scale study.

## CONCLUSIONS

The problem of depression and alcohol consumption is increasing in Nepal. Psychiatric comorbidity was found to be common in alcohol dependent people among whom depression was found to be most common followed by psychosis, anxiety, seizure disorder and OCD. The study showed that depression is common with Alcohol dependence. The likeliness is higher to be found among younger patients between the age group 30-45.
